# Message from the editors-in-chief

**DOI:** 10.1038/s41538-017-0004-2

**Published:** 2017-10-30

**Authors:** Pingfan Rao, Sharon P. Shoemaker

**Affiliations:** 1 Zhejiang Gongshang University, Hangzhou, China; 2 California Institute of Food and Agricultural Research, University of California Davis, Davis, CA 95616 USA

**Keywords:** Chemical biology

Food is vital to life. Of all the exogenous substances that interact with the human body, food is by far the most important in terms of quantity, complexity, mode of interaction, frequency, and hence its impact on human health and wellbeing. While environmental issues are at the top of today’s social agenda, food in all its forms and manifestations continues to demand the attention of scientists, policy makers, and the public. Although food science is sometimes confused with nutrition science, food science is unique in that it represents the penultimate step in the food value chain where nutrients are transformed into the safe, convenient, and wholesome foods that consumers desire.

While the contributions of food science to society and civilization may sometimes be taken for granted, no one can deny that our lives on this planet are made better by the foods and beverages we consume daily. Beginning with those early technologies that transformed wheat into bread, grapes into wine, and milk into cheese, this steady march of progress in food science can be seen all around us and in all its forms. Whether its school lunches for children, ready-to-use-therapeutic nutrition for the hungry and malnourished or medical foods for the sick and terminally ill, the handprint of food science is ever-present. It is the combination of discovery research and technology that enables food science to generate valuable knowledge beyond the field into other disciplines such as neuroscience, genomics, nanotechnology, health, and informatics.

Perhaps food science’s most enduring contribution to humankind is the development of delivery platforms for vital nutrients and energy to support those critical physiological systems of the body. Although there is still much to be learned about how this is accomplished, food science working in conjunction with other disciplines is making great strides toward this end. After nearly of century of research focusing on the peripheral blood system as the primary site for nutrient delivery, we now know that food, with all of its attendant nutrients, has the potential to interact with the 10–100 trillion symbiotic microbes that constitute the human microbiota and whose genes make up the microbiome, also called our “second genome.” The implications of this important finding are not fully understood at this time but they are sure to shift how we view nutrients and non-nutritive molecules passing through the alimentary canal. As an example, for nearly 15 years, the public has expressed concern over acrylamide, a common byproduct of baking. While considered a Group 2A carcinogen in small animal studies, acrylamide has yet to be proven to cause cancer in humans. With its ability to synthesize, degrade, and modify compounds in the gut, the human microbiota may be serving as a modulator and modifier of environmental inputs like acrylamide affecting how these inputs interact with the genome. Such interactions will require food science researchers to expand their scope of study from single molecules to more complex and dynamic molecular systems. This also holds true for food processing where foods processed differently, hence lead to different health impacts. Viewing diet-genome-microbiome interactions as a complex system should help researchers and the public to better understand the important difference in biological equivalency and bioavailability between the regular processed foods and specially designed food matrices for drug and nutraceutical delivery. By expanding the frontiers of science on food, researchers should be able address the public’s most common concerns and questions.

Toward this end, Nature Partner Journal (NPJ) has launched *Science of Food*, its first journal focusing on food science and technology. As editors-in-chief, we are encouraging submissions of original research articles, reviews, perspectives and editorials on cutting-edge advances in understanding the complex relationships between food, its constituents and how these factors relate to the nutritional, biological, chemical, and health sciences. Our vision is to approach food science holistically, from production, processing, and packaging to the consumer’s health outcome. The editors and reviewers will encourage technologies that are sustainable whenever possible and take a balanced and impartial view on research findings regardless of how the food is produced—conventionally, organically, or by genetic modification (GM).

We believe the broad scope of the journal will stimulate discussion of food science beyond its generally accepted parameters to include high impact, high quality research, and engineering that will help shape future directions for food science research as our planet approaches 10 billion people by the year 2050. The *Science of Food* will highlight those transdisciplinary discoveries and insights that will enable the food industry to create products with more predicable physiological effects and with qualities and characteristics suitable for extreme environments such as interplanetary space travel. These are just some of the ways that the *Science of Food* can help society meet its grand challenge of a sustainable, safer, healthier, prosperous, and peaceful world.

